# Non-Financial Support Provided to Parents in Stepfamilies: Empirical Examination of Europeans 50+

**DOI:** 10.3390/ijerph18105151

**Published:** 2021-05-13

**Authors:** Małgorzata Kalbarczyk

**Affiliations:** Faculty of Economic Sciences, University of Warsaw, Dluga Street 44/50, 00-241 Warsaw, Poland; mkalbarczyk@wne.uw.edu.pl

**Keywords:** stepchildren, care, SHARE, reciprocal exchange, kin selection

## Abstract

The aging of the population, coupled with increasing divorce and remarriage rates, are changing the structure of potential non-financial support for older parents. The purpose of this study was to examine support provided to parents aged 50+ in stepfamilies and to determine if the difference existed between help provided by natural children and stepchildren. The primary objective was to investigate whether blood ties were a significant determinant of the support if the quality of the relationship between the parent and a natural child or a stepchild was taken into account. The secondary objective was to answer the question to what extent the reciprocal exchange motive of support was observed in stepfamilies. The probability of non-financial support from children and stepchildren was estimated based on the sixth wave of the SHARE (Survey on Health, Ageing, and Retirement in Europe) database for European countries. Children in stepfamilies provided less non-financial help to parents than those in intact families. Stepchildren were less likely to be in stepparents’ social networks, and stepparents provided less help with childcare for grandchildren than they did to their biological children. Relationship closeness and looking after grandchildren increased the probability of non-financial support to older parents, regardless of whether the donor was a natural child or a stepchild.

## 1. Introduction

Rising divorce rates and falling marriage rates in Europe are changing the structure of the family [1,2,3]. Since 1965, the crude marriage rate decreased by about 50%, and, at the same time, the crude divorce rate doubled [4]. As a result of these demographic changes, the number of stepfamilies, defined as the union of two adults where at least one partner has a child from a previous relationship [5], is increasing globally [6,7,8]. Based on a microsimulation demographic model, Wachter [9] forecasted a decline in the number of biological kin and an increase in stepkin during the twenty-first century. Nowadays, the highest proportions of stepfamilies are found in the United States and Western European countries. In Sweden, 27% of children under 18 live with a stepparent; in Finland, Germany, and the UK, 25% of parents who have a child are separated, and most of them form new partnerships; 42% of Americans declare to have at least one step relative, and these percentages continue to rise [10].

The process of population aging increased the number of people requiring support in their old age. The current developments regarding both divorce and remarriage rates had an impact on the family structure of older people and, consequently, the availability of potential donors of support [11,12]. The family network of the elderly might be expanded due to the presence of stepchildren and more extensive stepfamilies, which would translate to greater help among larger families [9,13,14,15]. However, not all results of previous studies clearly indicated that an expanded family network (even without stepchildren) increased informal support to older parents. Firstly, in large families, bonds and closeness between family members might be weaker, and secondly, the responsibility for providing help was more dispersed among multiple family members, which might result in less help [16,17]. Wimer et al. [18] showed that in the US, although stepkin increased the family size, the likelihood of transfer of time assistance in stepfamilies was lower.

The two basic theories explaining intergenerational transfers are kinship selection and the reciprocal exchange theory. The kinship selection theory [19] assumes that individuals will prefer to invest in their closely related kins rather than unrelated individuals. The reciprocal exchange [20] can explain transfers between non-kins when the initial transfer is rewarded by return help from the person who previously received support. The motivation and decisive factors for providing help to stepparents by stepchildren may not be the same as for adult children and their elderly parents. Kinship selection can be a major motive for help from biological children to their older parents but may not be applicable to intergenerational transfers between stepchildren and stepparents. Reciprocal exchange can be the incentive for providing help in stepfamilies [21]; for example, help given to stepparents may reciprocate the assistance with childcare for grandchildren later on in life. Research conducted on US databases, which analysed the help provided to older parents by children, showed that such help was less often given by stepchildren [17,18,22]. Similarly, research on support provided by older parents to children or grandchildren showed that parental help occurred more often when biological children or grandchildren were supported. Eggebeen [23] showed that an increase in the proportion of stepchildren had a negative influence on the propensity for giving assistance to children. Tanskanen et al. [24] and Coall et al. [25] showed that grandparents were less likely to provide childcare assistance to stepchildren, which was in line with the kin selection theory.

In both theories, relationship quality and emotional closeness between family members played an important role in intergenerational support. Children provided more support when relationships were close, compared to when they were distant [22,26,27]. Research showed that relationships between stepparents and adult stepchildren tended to be characterized by weaker emotional closeness than relationships with biological children [28,29,30]. However, when a close relationship between the stepparent and stepchildren occurred, it would increase the likelihood of behaviour between the kins. Close bonds with adult stepchildren might even lead to more help given to the stepparent than in the case of parents who did not have a close relationship or had severed relationships with their biological children [22].

The aim of this study was to identify the determinants of receiving support by Europeans over the age of 50 in stepfamilies while assessing the differences between non-financial help given by natural children and stepchildren. The secondary objective was to determine whether factors other than blood ties were significant in stepfamilies in the context of providing non-financial support. In particular, the quality of the relationship between the parent and the natural child or stepchild and the reciprocal exchange motive of support might have an influence. The research questions are:

Q1: Do stepchildren provide less non-financial help to parents than natural children in stepfamilies?

Q2: Is the likelihood of providing non-financial help to parents in stepfamilies greater for children with close relationships, regardless of whether it is a natural child or stepchild?

Q3: Is the probability of receiving non-financial help by parents in stepfamilies greater from children whose children are looked after by the grandparents, regardless of whether they are natural grandchildren or step grandchildren?

Understanding to what extent stepchildren could be donors of informal, non-financial help for older stepparents in the light of the ongoing demographic changes was crucial for both the point of view of older individuals as well as for future changes in social policies of countries. Prior research on support among European stepfamilies’ members was scarce, and neither provided such a broad overview of multiple European countries nor did it include all the above-mentioned factors and comparisons.

## 2. Material & Methods

In the analysis, the SHARE (Survey on Health, Ageing, and Retirement in Europe) database was used. The SHARE is a biennial panel study conducted on a representative sample of people aged 50 years or older and their partners across European countries [31]. The study brings together many disciplines, including demography, economics, epidemiology, and psychology. The data contains information regarding the respondents’ family composition, social network, functional capacity as declared by the respondents, and support received and given. The presented analyses were based on the wave sixth release 7.1.0, which was conducted in 2015. The sixth wave was the last wave that included detailed information on non-financial support, children, and social networks alike.

The empirical analysis included seventeen European countries (Israel was excluded) grouped into four geographical regions that differed with regard to intergenerational relations and welfare systems [32,33]. The groups were as follows:

- northern countries: Denmark, Sweden
- western countries: Austria, Germany, France, Switzerland, Belgium, Luxemburg
- southern countries: Spain, Italy, Greece, Slovenia, Croatia, Portugal
- eastern countries: Poland, Czech Republic, Estonia.

The sample covered 58,242 respondents after excluding those who did not have children or did not provide information about them. The data was complemented with information provided by respondents in the previous waves of the survey (for example regarding children). Respondents with missing data, after completing, were excluded from the analysis. For each respondent, the information on their family composition was available, including the relation to the child, whether it was a natural child of the respondent and the partner, a natural child of the respondent but not the partner, a natural child of the partner but not the respondent. In the sample, 3330 respondents declared to have at least one stepchild. The econometric analysis was conducted at the level of a parent and child dyad. The database included 138,490 such dyads, of which 5952 were between a stepparent and a stepchild. In order to focus on the factors influencing the probability of receiving help from children in stepfamilies, the sample of the econometric analysis was limited only to parent and child dyads in stepfamilies. The first model included all children in stepfamilies. Then, to see the exact differences between stepchildren and natural children in stepfamilies, a second model including only stepparent and stepchild dyads and a third with parent and natural child dyads in stepfamilies were estimated.

For each natural child and stepchild, the parents indicated whether they received informal help from that child. Information about informal care was based on responses to these two questions:

“Thinking about the last twelve months, has any family member from outside the household, any friend or neighbour given you any personal care or practical household help?” (kind of help included: dressing, bathing or showering, eating, getting in or out of bed, using the toilet, with home repairs, gardening, transportation, shopping, household chores or help with paperwork, such as filling out forms, settling financial or legal matters).

“Is there someone living in this household who has helped you regularly during the last twelve months with personal care, such as washing, getting out of bed, or dressing?”

Respondents indicated, for outside and inside the household care, three people who provided help, and if it was a child, they indicated exactly which child it was. The dependent variable was support from a child, which was a binary variable with the value one if the given child was providing help and zero otherwise. The regression models were fitted with logistic regressions. The control variables were the respondents’ sex, age, education (in years), household size, and place of residence (rural or urban), and country group variable. In order to control people, who required more help due to their physical condition, the model took into account the number of ADL (Activity of Daily Living) difficulties and the number of IADL (Instrumental Activity of Daily Living) difficulties. When analysing help for older parents provided by children in stepfamilies, it was important to take into account the characteristics of the child. From the data available in the SHARE database, the child’s gender, occupation status (working or not), the geographical distance between the child and the parent (distance up to 5 km (including co-residence), 5–25 km, 25–100 km, 100 km, and over), and whether the parent looks after the grandchildren of a given natural child or stepchild were the selected variables. Apart from child characteristics, it was important to understand how the presence of other children and help received from them influenced children’s decisions to provide help. Therefore, the number of natural children and a variable indicating whether the parent received help from them were included in the second model. In the first and third models, the total number of children was included.

Including the information on whether the child was in the parents’ social network was essential for testing the research questions. In the SHARE database, respondents were asked the following question:

“Most people discuss with others the good or bad things that happen to them, problems they are having, or important concerns they may have. Looking back over the last 12 months, who are the people with whom you most often discussed important things? These people may include your family members, friends, neighbours, or other acquaintances. Please refer to these people by their first names.”

Each respondent reported up to seven such people who were in their social network, and it was possible to identify if a particular child was in this network. A variable indicating whether a given child was in the parents’ social network was included in the second and third models. In the first model, an interaction between the variable indicating whether the child was in the parents’ social network and the variable determining whether the child was natural or stepchild was included.

Due to the fact that each parent could have several children, the data in logistic regressions were clustered. This clustering method took into account nonindependence of each dyad of the same parent.

## 3. Results and Discussion

### 3.1. Descriptive Findings

Parents with natural children were the majority of the SHARE sample (94%), and parents with at least one stepchild accounted for only about 6 percent. The differences were visible between the groups of countries. In Western and Eastern Europe, the percentages were similar (6.6% and 7.1%, respectively). Significant differences could be seen in northern countries, where this percentage was higher, 12.7%, and in southern countries, where the percentage of stepfamilies was the lowest at 1.9%. The average number of children in stepfamilies was 1.6, and it was higher than in families without stepchildren, where the average was 1.1.

In stepfamilies, the share of stepchildren was higher than natural children and equalled 64 percent. Stepchildren were much less often included in stepparents’ social networks than natural children (9% versus 33%, Table 1). Stepparents were also less likely to look after their step grandchildren. In the case of step grandparents, only 7.7% looked after their grandchildren, while for natural grandparents, this percentage was higher and amounted to 12.6% (Table 1). These results were in line with the existing literature [24,25,30,34]. Less inclusion in the social network and less contribution to the care of grandchildren by stepparents could result both from stronger emotional bonds and existing blood ties with natural children. It seemed that the geographical distance between child and parent was not the cause. The differences in geographical distance between step- and natural children were not significant. The results showed that 52.6% of stepchildren and 55.8% of natural children lived within 25 km of the parents. Additionally, percentages of children living more than 100 km away were also comparable (26.7 and 25.9 for step- and natural children, respectively, Table 1).

In the entire population of parents, more than one out of ten declared that they received support from children outside or inside the household. In stepfamilies, the support provided by children was less frequent (7.5%), which confirmed the results obtained by Wimer et al. [17] or Pezzin and Schone [18]. Characteristics of parents in stepfamilies who received support showed that they were older, had more children, and, on average, had more ADL or IADL limitations than those who were not recipients of help (0.73 and 0.28 for IADL, and 0.47 and 0.17 for ADL, respectively; Table 2). Additionally, those living in eastern countries received support most often.

Although the share of stepchildren was higher than natural children, natural children accounted for 58% of all support providers in stepfamilies. Members of parents’ social network contributed most to the support in stepfamilies (53.7 percent of natural caregivers and 31.6 percent of step caregivers were in the parents’ social network, while in the group of children who did not provide the support, these percentages were 32.1 and 8.6, respectively; Table 1). At the same time, the support from children in stepfamilies decreased as the geographical distance extended both for natural children and stepchildren. The percentages of parents looking after their grandchildren were significantly higher among natural children and stepchildren who were help providers. It was worth noting that features that distinguished children providing help to their parents and those who did not, were not dependent on being a step- or natural child in stepfamilies.

### 3.2. Logistic Regression Model

Three logistic regression models showed the determinants of the probability of non-financial support received from children in stepfamilies (Table 3). Children who were in the social network were more likely to provide support to parents. In the first model, the interaction between the social network variable and being a natural or a stepchild was statistically significant. Being in the parents’ social network, for both a natural child and a stepchild, increased the probability of providing help in relation to a natural child who was not in the parents’ social network. The coefficient was even higher for a stepchild than a natural child. On the other hand, comparing a natural child and a stepchild who were not included in a parents’ social network, the stepchild was less likely to provide help to the parent than the natural child. Older parents, who supported their children by providing childcare for the grandchildren, were more likely to receive help from children who were the parents of these grandchildren. Interestingly, the influence of caring for grandchildren on the probability of non-financial support was also visible among stepparents and stepchildren, where kinship altruism could not be the explanation. It was worth noting here that among people who did not look after their grandchildren, there were both those who did not want to or could not take care of them and those who did not have grandchildren or the grandchildren no longer need care. However, this did not affect the observed result for step- and natural children that the motive for helping the elderly parents did not have to be kinship altruism.

Consequently, other motives of providing non-financial support in stepfamilies beyond kinship altruism based on the kinship selection theory might be observed. Involvement in a close relationship between the child and the parent measured by the inclusion of the child in the parents’ social network as well as the engagement of grandparents in taking care of their grandchildren were factors that positively influenced the probability of non-financial support, regardless of whether it was provided by a step- or natural child. This result was in line with Ganong and Coleman [22], who pointed out that the relationship quality was important in attributing responsibilities to help both parents and stepparents in the US. However, for stepchildren, it was more difficult to enter stepparents’ social network (this result was consistent with the literature: [28,30]), and stepparents were less likely to get involved in looking after the grandchildren (this was also in line with other research: [24,25]) but if it happened, the probability of returned help would be on a similar level for step- and natural children in stepfamilies.

As for child characteristics, the probability of receiving non-financial help from children diminished as the geographical distance between child and parent increased, which was in accordance with the literature [35,36]. Parents with stepchildren living at least 5 km away were less likely to receive support. For natural children, statistically significant differences were observed when the distance was over 100 km. In the case of stepchildren, the lack of blood ties meant that the geographical distance had to be closer for the exchange of support to take place. According to the results, female children were less likely to provide support in stepfamilies. A significant effect could be observed for both natural children and stepchildren. This finding was not in line with the literature (for example [37]) but could be explained, for example, by various forms of support being under consideration, i.e., not only personal care but also practical help, both at home and in filling in documents, which might more often be done by men. Additionally, the fact that the amount of help was not measured and the data only indicated whether a given child provided help might have an impact on the result. Children’s occupation status was not statistically significant.

As for the parent characteristics, we found that parents with more ADL and IADL limitations received support more frequently (effect for ADL and IADL was statistically significant in the first model, in the second model only for ADL, and in third only for IADL). Parents’ education and place of living were not statistically significant. Mothers received help more frequently from natural children in stepfamilies, and being an older stepparent increased the probability of receiving support from a stepchild. The total number of children did not have a statistically significant influence on the probability of receiving help by a parent. However, a higher number of natural children statistically significantly reduced the probability that a stepchild would provide non-financial help. On the other hand, non-financial support from other children in the family increased the probability that a given child would help. This might be explained by the prevailing values and the way of upbringing children in a given family. Children from homes where helping other family members was highly valued tended to help their parents or stepparents alike. Country effects were significant for Eastern European countries, where the probability of support from natural children and stepchildren in stepfamilies was higher than in Northern European countries. Additionally, with regards to helping received from stepchildren, the probability was higher also in Northern European countries. Due to the cultural background in the Eastern countries, informal non-financial help provided mainly by children was much more frequent than in the Northern and Western European countries.

The study had some limitations, mostly related to the SHARE database itself. First, we only analysed the fact of receiving support without information about the amount of help. The help as such, defined as zero-one-variable, did not provide full information about the involvement in support. It was possible that both natural children and stepchildren helped their parents in stepfamilies, but the former spend much more time doing this. Another limitation concerned the sample size of parents in stepfamilies. The SHARE database included people over 50 years old. The average age of the parents was around 68, while the stepparents were younger. Stepchildren were rare among the oldest respondents. Due to the growing number of divorces and remarriages, the percentage of stepfamilies will increase in the following years, which would allow the study to be repeated on a larger sample. This could lead to the identification of a greater number of statistically significant determinants of receiving help.

## 4. Conclusions

The presented study showed that children in stepfamilies provided less help than those in intact families. Non-financial support within stepfamilies was more often provided by natural children. It was also worth noting that stepchildren were less likely to be in stepparents’ social networks and received less help from them in looking after the grandchildren, as compared to biological children. However, both these factors, i.e., the closeness of the relationship and caring for grandchildren, motivated natural children and stepchildren alike to provide non-financial assistance to older parents or stepparents. Investing in a good and close relationship between a stepchild and a stepparent was less common and probably more difficult due to the lack of blood ties. However, it might result in better mutual non-financial support. In the face of demographic changes leading to an increase in the number of elderly people with stepchildren in the family, further research into the relationship and exchange of non-financial support between stepparents and stepchildren will help determine the scale and type of support that older people can receive.

## Figures and Tables

**Table 1:** Descriptive statistics of children in stepfamilies, by giving non-financial support.

	Natural Children			Stepchildren		
	Total	Support Giver	Not Support Giver	Total	Support Giver	Not Support Giver
Female (%)	50.52	45.37	50.77	49.65	40.44	49.86
Not working (%)	26.54	25.93	26.57	23.52	18.38	23.64
Being in parents’ social network (%)	33.04	53.70	32.05	9.15	31.62	8.62
Co-residing	13.40	8.06	13.67	9.36	12.71	9.28
Distance <5 km	20.17	36.02	19.36	18.77	33.90	18.40
Distance 5–25 km	22.26	23.12	22.21	24.43	22.80	24.46
Distance 25–100 km	18.30	17.74	18.32	21.06	16.95	21.16
Distance >100 km	25.88	15.05	26.43	26.38	13.56	26.69
Receiving grandchildren babysitting care (%)	12.56	21.76	12.12	7.67	19.85	7.38

Note: Sample covers children in stepfamilies (with at least one stepchild), natural children *n* = 4697, stepchildren *n* = 5948. Source: Author’s own elaboration based on SHARE wave 6, release 7.1.0.

**Table 2:** Descriptive statistics of respondents with at least one stepchild, by non-financial support received.

	Total	Non-Received Support	Received Support
Average–age	64.51	64.36	66.31
Average–number of ADL	0.20	0.17	0.47
Average–number of IADL	0.32	0.28	0.73
Average–household size	2.30	2.31	2.19
Average–education (in years)	12.05	12.06	11.95
Average–number of natural children	0.74	0.73	0.90
Female (%)	47.72	47.37	52.00
Rural (%)	34.87	35.00	33.20
Western Countries (%)	37.51	38.57	24.40
Northern Countries (%)	26.34	26.30	26.80
Southern Countries (%)	12.16	12.79	4.40
Eastern Countries (%)	23.99	22.34	44.40

Note: respondents with at least one stepchild *n* = 3330; Source: Author’s own elaboration based on SHARE wave 6, release 7.1.0.

**Table 3:** Coefficients in the logistic regression models for non-financial support given by children in stepfamilies.

Determinants of Non-Financial Support Given by Children in Stepfamilies	Model 1 Children in Stepfamilies	Model 2 Stepchildren	Model 3 Natural Children in Stepfamilies
Child’s gender	−0.55 ***	−0.75 ***	−0.44 **
Child’s occupation status (ref.: working)	0.12	−0.07	0.30
Child’s availability (ref.: <5 km)			
5–25 km	−0.49 ***	−0.52 **	−0.38
25–100 km	−0.57 ***	−0.63 **	−0.39
>100 km	−1.00 ***	−1.13 ***	−0.88 ***
Child in social network		1.42 ***	0.84 ***
Interaction social network (SN) & being stepchild (ref: not in SN & natural child)			
in SN & natural child	0.78 ***		
not in SN & stepchild	−0.62 ***		
in SN & stepchild	0.86 ***		
Grandchildren babysitting	0.71 ***	1.08 ***	0.41 **
Household size	−0.54 ***	−0.52 **	−0.55 ***
Number of natural children		−0.41 ***	
Support from natural children		2.28 ***	
Number of children	0.03		0.06
Support from stepchildren			1.82 ***
Age	0.005	0.02 *	−0.01
Education in years	0.0002	−0.007	0.006
Female (ref.: male)	0.23	−0.07	0.61 ***
ADL limitations	0.21 *	0.29 *	0.04
IADL limitations	0.15 *	0.02	0.24 **
Rural (ref.: town)	−0.002	−0.14	0.18
Country effects (ref: Western countries)			
Northern countries	0.36	0.55 *	0.25
Southern countries	−0.44	−0.63	−0.31
Eastern countries	1.25 ***	1.35 ***	0.99 ***
Constant	−2.77 ***	−3.97 ***	−3.19 **
Number of observations	8217	4644	3589
Presudo-R^2^	0.13	0.22	0.13

Notes: statistical significance levels: *** *p* < 0.01, ** *p* < 0.05, * *p* < 0.1. Source: Author’s own analysis based on SHARE wave 6, release 7.1.0.

## Data Availability

This paper uses data from SHARE Waves 1, 2, 3, 4, 5, 6, 7 and 8 (DOIs: 10.6103/SHARE.w1.710, 10.6103/SHARE.w2.710, 10.6103/SHARE.w3.710, 10.6103/SHARE.w4.710, 10.6103/SHARE.w5.710, 10.6103/SHARE.w6.710, 10.6103/SHARE.w7.711, 10.6103/SHARE.w8cabeta.001), see Börsch-Supan et al. (2013) for methodological details.(1). The SHARE data collection has been funded by the European Commission through FP5 (QLK6-CT-2001-00360), FP6 (SHARE-I3: RII-CT-2006-062193, COMPARE: CIT5-CT-2005-028857, SHARELIFE: CIT4-CT-2006-028812), FP7 (SHARE-PREP: GA N°211909, SHARE-LEAP: GA N°227822, SHARE M4: GA N°261982, DASISH: GA N°283646) and Horizon 2020 (SHARE-DEV3: GA N°676536, SHARE-COHESION: GA N°870628, SERISS: GA N°654221, SSHOC: GA N°823782) and by DG Employment, Social Affairs & Inclusion. Additional funding from the German Ministry of Education and Research, the Max Planck Society for the Advancement of Science, the U.S. National Institute on Aging (U01_AG09740-13S2, P01_AG005842, P01_AG08291, P30_AG12815, R21_AG025169, Y1-AG-4553-01, IAG_BSR06-11, OGHA_04-064, HHSN271201300071C), and from various national funding sources is gratefully acknowledged (see www.share-project.org). Data are available on www.share-project.org.
